# Optimization of Glycolipid Synthesis in Hydrophilic Deep Eutectic Solvents

**DOI:** 10.3389/fbioe.2020.00382

**Published:** 2020-05-05

**Authors:** Rebecca Hollenbach, Benjamin Bindereif, Ulrike S. van der Schaaf, Katrin Ochsenreither, Christoph Syldatk

**Affiliations:** ^1^Institute of Process Engineering in Life Sciences II: Chair of Technical Biology, Karlsruhe Institute of Technology, Karlsruhe, Germany; ^2^Institute of Process Engineering in Life Sciences I: Chair of Food Process Engineering, Karlsruhe Institute of Technology, Karlsruhe, Germany

**Keywords:** glycolipid, deep eutectic solvents, enzymatic synthesis, mass transfer, viscosity, *Candida antarctica* lipase B

## Abstract

Glycolipids are considered an alternative to petrochemically based surfactants because they are non-toxic, biodegradable, and less harmful to the environment while having comparable surface-active properties. They can be produced chemically or enzymatically in organic solvents or in deep eutectic solvents (DES) from renewable resources. DES are non-flammable, non-volatile, biodegradable, and almost non-toxic. Unlike organic solvents, sugars are easily soluble in hydrophilic DES. However, DES are highly viscous systems and restricted mass transfer is likely to be a major limiting factor for their application. Limiting factors for glycolipid synthesis in DES are not generally well understood. Therefore, the influence of external mass transfer, fatty acid concentration, and distribution on initial reaction velocity in two hydrophilic DES (choline:urea and choline:glucose) was investigated. At agitation speeds of and higher than 60 rpm, the viscosity of both DES did not limit external mass transfer. Fatty acid concentration of 0.5 M resulted in highest initial reaction velocity while higher concentrations had negative effects. Fatty acid accessibility was identified as a limiting factor for glycolipid synthesis in hydrophilic DES. Mean droplet sizes of fatty acid-DES emulsions can be significantly decreased by ultrasonic pretreatment resulting in significantly increased initial reaction velocity and yield (from 0.15 ± 0.03 μmol glucose monodecanoate/g DES to 0.57 ± 0.03 μmol/g) in the choline: urea DES. The study clearly indicates that fatty acid accessibility is a limiting factor in enzymatic glycolipid synthesis in DES. Furthermore, it was shown that physical pretreatment of fatty acid-DES emulsions is mandatory to improve the availability of fatty acids.

## Introduction

Glycolipids are a class of biosurfactants that have been claimed to be non-toxic (Hirata et al., 2009), readily biodegradable (Baker et al., 2000; Hirata et al., 2009; Lima et al., 2011), and therefore, less harmful to the environment than the petrochemically produced ones (Dörjes, 1984; Poremba et al., 1991; Lima et al., 2011; Johann et al., 2016). Glycolipids are of special interest to the pharmaceutical industry, e.g., as bioavailability enhancers (Perinelli et al., 2018), and for the food industry, since e.g., sucrose fatty acid esters are approved as food additives (European Parliament, 2014; Younes et al., 2018). Apart from these applications, they can also be used in the detergent industry, textile industry and cosmetic industry, as well as in the agrochemical and the petroleum industry (Shete et al., 2006).

The enzymatic synthesis of sugar surfactants is well established in volatile organic solvents (Castillo et al., 2003; Šabeder et al., 2006), but sugar solubility is limited in this system (Flores et al., 2002). Hydrophilic deep eutectic solvents (DES) have been reported as an alternative characterized by good sugar solubility and, in addition, non-volatility and non-flammability. DES consist of a hydrogen bond acceptor and a hydrogen bond donor (Abbott et al., 2006; Zhang et al., 2012; Durand et al., 2016). Hydrophilic DES consisting of choline as hydrogen bond acceptor and urea or glucose as hydrogen bond donor are proven to be readily biodegradable and have low cytotoxicity (Radošević et al., 2015; Wen et al., 2015; Mbous et al., 2017). If glucose is used as a hydrogen bond donor, it serves simultaneously as substrate for the enzymatic reaction. The synthesis of sugar surfactants in DES was first described by Pöhnlein et al. (2015). In 2018, this process was first conducted entirely based on lignocellulosic materials (Siebenhaller et al., 2018). To date, there is only one study to be found that includes a quantitative analysis of synthesis in a DES containing system. In that study, Zhao et al. (2016), investigated various biphasic systems of an organic solvent with 10% of different choline-based DES, using urea, acetamide, glycerol or ethylene glycol as the hydrogen bond donor. Low or negligible glycolipid yields were reported (Zhao et al., 2016).

**FIGURE 1:**
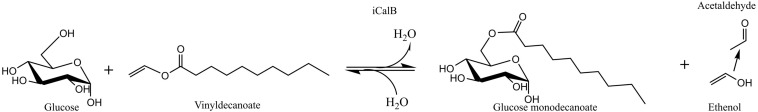
Reaction scheme of enzymatic synthesis of glucose monodecanoate.

Indeed, the evaluation of the limiting factors or optimization of glycolipid synthesis in DES has not been reported so far, although the high viscosity of DES is considered to be a major problem for DES applications (Dai et al., 2013), implying limited mass transfer of reactants. The investigation of different agitation rates without changing any other reaction parameter has been reported as suitable for the determination of an external mass transfer limitation (Zhang et al., 2004; Gonçalves et al., 2008; Ülger and Takaç, 2017). Hence, in this study, external mass transfer was investigated by using the enzymatic synthesis of glucose monodecanoate as a model reaction (Figure 1). In order to evaluate the influence of different reaction parameters and to identify the limitations of glycolipid synthesis in DES, and due to the challenge, posed by low concentrations on the analytics, a sensitive high performance liquid chromatography (HPLC) method with evaporative light scattering detection was developed for the analysis of glycolipids in this study. However, the high viscosity of DES reaction systems prevents a direct HPLC analysis, making sample extraction necessary. Therefore, extraction efficiency of three different extractants was also evaluated.

Two different hydrophilic DES, which were previously described in literature, were used for glycolipid synthesis. One consists of choline chloride and urea (ChCl:U), while the other contains choline chloride and glucose (ChCl:Glc). In the latter case, glucose simultaneously contributes as part of the solvent and substrate for the reaction. Unlike organic solvents, sugar solubility is not restricted in both hydrophilic DES, but the accessibility of the second substrate, the hydrophobic fatty acid, might be limited due to the formation of fatty acid-DES emulsions. Hence, mean droplet size of the emulsion was determined as a measure for fatty acid distribution, and availability and the impact on the reaction velocity was investigated. Finally, with the results obtained, a strategy to optimize the reaction can be developed.

## Materials and Methods

### Materials

All chemicals were acquired from Carl-Roth (Germany) if not stated otherwise. All solvents were in HPLC grade. Lipase B from *Candida antarctica*, immobilized on acrylic resin (iCalB), was purchased from Strem Chemicals (Strem chemicals Europe, Germany). Vinyl decanoic acid was acquired from Tokyo Chemical Industry Co., Ltd. (TCIEurope, Belgium). 6-Decanoyl-D-glucose was purchased from Sohena (Germany). Double distilled water (0.005 mS) was obtained using a Purelab flex water system from Elga LabWater (Celle, Germany).

### Preparation of DES

Two different DES based on choline chloride were used in this study: choline chloride urea (ChCl:U) and choline chloride glucose (ChCl:Glc). For the preparation of ChCl:U, choline chloride and urea were mixed in a molar ratio of 1:2 (n:n) and 5% (v/v) of double distilled water was added. For ChCl:Glc, choline chloride, glucose and water were mixed in a ratio of 5:2:5 (n:n:n). The mixtures (200 g for each DES) were heated in a sealed glass bottle to a temperature of 90°C and stirred at 600 rpm using a NeoMag magnetic stirrer from neoLab (Heidelberg, Germany) for 2 h until a colorless fluid was obtained. Then, the DES were allowed to cool to room temperature.

### Ultrasonic Pretreatment

Samples of 10 mL DES containing 0.5 M vinyldecanoate were sonicated for 5 min with 60% amplitude and a cycle of 20 s pulsing and 30 s pause in 50 mL tubes. A Sonopuls HD 3100 ultrasonic homogenizer from Bandelin (Berlin, Germany) equipped with a MS 72 probe was used at a frequency of 20 kHz (with an energy input of 4.654 kJ). The probe was set to an immersion depth of 1.5 cm. During sonication, the samples were cooled in a water bath after which they were immediately used for synthesis.

### Synthesis of Glycolipids (Standard Reaction)

Glycolipid synthesis was carried out in 5 mL tubes (Eppendorf AG, Hamburg, Germany) filled with 2 mL DES. Vinyl decanoate was added to a final concentration of 0.5 M to both DES, while glucose (final concentration 0.5 M) was added only to ChCl:U. Finally, by adding 20 mg/mL iCalB the reaction was started. The tubes containing 2.5 g of reaction mixture, were mixed in a rotator with a vortex mixer (program U2) from neoLab (Heidelberg, Germany) at working conditions of 90 rpm and 50°C. The reaction time varied between 4 h (to determine the initial reaction velocity) and 24 h (to calculate the total yield). At each time point of interest, three tubes (for triplicate measure) were collected and totally processed for further analysis.

### External Mass Transfer Limitation Test

In order to examine external mass transfer limitation, the agitation rates were varied from 30 rpm to 60 and 90 rpm with a reaction time of 4 h. All other reaction conditions were kept constant.

### Influence of Fatty Acid Concentration

To address the optimal fatty acid concentration for the reaction, different fatty acid concentrations (0.25, 0.5, 0.75, and 1.0 M) were tested without varying any other reaction parameter.

### Synthesis With Ultrasonic Pretreatment

For the synthesis with ultrasonic pretreatment, 2 mL of each sonicated fatty acid-DES emulsion was filled into 5 mL tubes. In the case of ChCl:U, glucose (0.5 M) was supplemented. Finally, 20 mg/mL iCalB was added. The other reaction conditions were remained same as the standard reaction.

### Extraction of Glycolipids

For glycolipid extraction, 3.42 mL of extraction solvent and 1.1 mL of double distilled water were mixed with the reaction tube content (2.5 g) in a rotator with vortex mixer (program U2) at an agitation rate of 90 rpm. Temperature and time were set to 50°C and 20 min, respectively, after which the upper organic phase was collected for HPLC analysis.

Three different extraction solvents were tested under similar conditions as stated above, but the volume was halved (1.71 mL of extraction solvent and 0.55 mL of double distilled water per mL of DES): ethyl acetate (EtAc), dimethyl carbonate (DMC) and chloroform were tested as extractants. To evaluate the extraction performances, 5 mg of glucose decanoate was incubated in 1 mL of both DES for at least 5 h and afterward extracted and analyzed by HPLC-ELSD. The extraction efficiency was calculated as follows:

extractionefficiency[%]

$$=\frac{\mathrm{glucose}\,\mathrm{monodecanoate}\,\mathrm{content}\,\mathrm{measured}}{\mathrm{glucose}\,\mathrm{monodecanoate}\,\mathrm{content}\,\mathrm{added}}\times 100\%$$

### HPLC-Evaporative Light Scattering Detector (ELSD)

Glucose decanoate was determined by HPLC using a Kinetex EVO C18 (2.6 μm, 250 mm × 4.6 mm) column from Phenomenex (Aschaffenburg, Germany) with an accompanying guard column (4 mm × 3.0 mm ID) of the same phase, using an Agilent 1260 series liquid chromatograph (Waldbronn, Germany) equipped with a quaternary pump, an autosampler and a column oven. An evaporative light scattering detector (ELSD) from BÜCHI Labortechnik (Essen, Germany) was used for detection. The mobile phase, solvent A, was water and solvent B was acetonitrile. The flow rate was 1 mL/min and a gradient was used for separation of products and substrates: starting from 40% A-60% B, then 0–10 min a linear gradient up to 35% A-65% B, followed by another linear gradient from 10 to 15 min up to 25% A-75% B. This gradient was held for 5 min, followed by a reconditioning step of the column with 40% A-60% B for 5 min. The injection volume was set to 10 μL. The column was operated at 50°C. The detector was operated at 38°C with a gas flow (air) of 1.5 mL/min. The gain was set to 1.

### Viscosity Measurements

The viscosity of DES was measured using a Physica MCR 101 viscometer (Anton Paar, Ostfildern, Germany) with double gap geometry (DG26.7) and shear rates of 2-100 s^–1^. Measurements were conducted at temperatures of 20 and 50°C.

### Droplet Size Distribution Measurements

The droplet size distribution of vinyl decanoate in DES emulsions was characterized using a Horiba LA-940 laser diffraction particle analyzer from Retsch Technology GmbH (Haan, Germany). Refractive indices required for the calculation of the droplet size distribution by the built-in software were determined as 1.4362 for vinyl decanoate, 1.4971 for ChCl:U and 1.4981 for ChCl:Glc by an analogous Abbe refractometer AR4 (Krüss Optronic, Hamburg, Germany). Samples were measured directly after preparation.

### Statistical Analysis

Results are presented as mean ± standard deviation (*n* = 3). Statistical data analysis was performed by two-way ANOVA and Tukey test using the OriginPro 9.6 (version 2019) software. Results were considered significant if *p*-value was <0.05.

## Results

The main purpose of this study was to identify the limiting factors and therefore optimization potential for glycolipid synthesis in two hydrophilic DES requiring a reliable and sensitive quantification method. The first part therefore describes the development and evaluation of a glycolipid extraction method, as well as the quantification of the model substrate glucose monodecanote by HPLC-ELSD.

### Quantification of Glucose Monodecanoate

Glucose monodecanoate was successfully separated from glucose and vinyl decanoate using the developed HPLC-ELSD method (Supplemetary Figure S1). The retention times were 2.1 min for glucose, 2.68 min for glucose monodecanoate and 5.76 min for vinyl decanoate. Due to the low baseline noise and the peak resolution (Table 1), glucose monodecanoate can be quantified in a range between 0.015 μ mol/ml and 4.49 μmol/ml.

**TABLE 1:** Chromatographic and analytical characteristics of glucose monodecanoate analysis using HPLC-ELSD.

**Retention time (glucose monodecanoate)^a^**	**2.68–2.72 min**
Correlation coefficient (*R*^2^, *n* = 3)	0.9975
Equation of linear calibration	y = 9759.9x – 247.87
Linear range of calibration	0.06–4.49 μmol/mL
Resolution_*glucose* – *glucose monodecanoate*_ (*n* = 3)	7.7
Resolution_*glucose monodecanoate* – *decanoic acid*_ (*n* = 3)	29.5
Peak width^b^	0.039–0.054 min
Baseline noise (*n* = 3)	0.06 mV
Limit of detection (signal/noise = 3)	<0.0014 μmol/mL
Limit of quantification (signal/noise = 10)	0.0014 μmol/mL

*^*a*^Inter-day variance of retention time measured at 3 different days. ^*b*^Concentration 0.06–4.49 *μ* mol/mL.*

### Extraction Efficiency of Different Solvents

**FIGURE 2:**
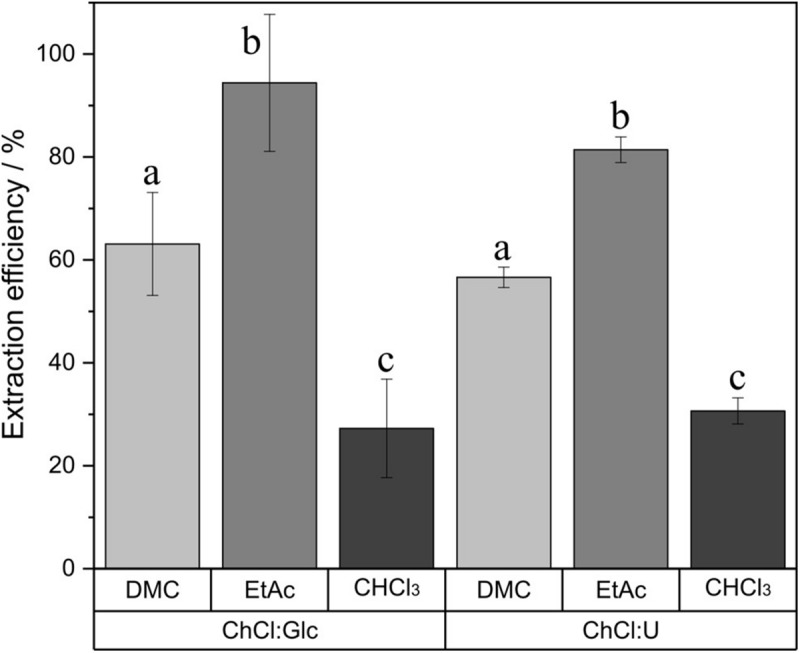
Comparison of three extraction solvents, dimethyl carbonate (DMC), ethyl acetate (EtAc) and chloroform (CHCl_3_), for glucose decanoate extraction from ChCl:Glc (ChCl:Glc:water, 5:2:5, n:n:n) and ChCl:U (ChCl:U, 1:2, n:n, 5% water). a, b, and c show statistically significant differences.

In order to provide a reliable extraction method for the quantification of glycolipids, three different extraction solvents were tested (Figure 2). In this regard, chloroform, EtAc and DMC were used to extract glucose decanoate from the DES, and their performance was evaluated. With all three solvents, a two-phase system was formed: a DES-water phase and an organic solvent phase containing the glucose monodecanoate. Chloroform was the worst extraction solvent with an efficiency of a single extraction of 30.6 ± 2.5% (ChCl:U) and 27.3 ± 9.6% (ChCl:Glc). In contrast, the solvents EtAc and DMC were statistically more effective with EtAc showing the highest yields of 81.4 ± 2.5% (ChCl:U) and 94.4 ± 13.3% (ChCl:Glc). Therefore, EtAc was chosen as an extractant for this study.

### External Mass Transfer Limitation Test

In order to investigate the effect of viscosity on glycolipid yield, the viscosity of both types of DES was measured. Both DES showed Newtonian behavior, with ChCl:U having a viscosity of 0.28 ± 0.03 Pa⋅s and ChCl:Glc of 1.41 ± 0.16 Pa⋅s at 20°C. For contrast, the viscosity of water is 0.001 ± 0.00 Pa⋅s. At 50 C, the viscosity of ChCl:U is 0.053 ± 0.0004 Pa⋅s and that of ChCl:Glc is 0.17 ± 0.002 Pa⋅s.

**FIGURE 3:**
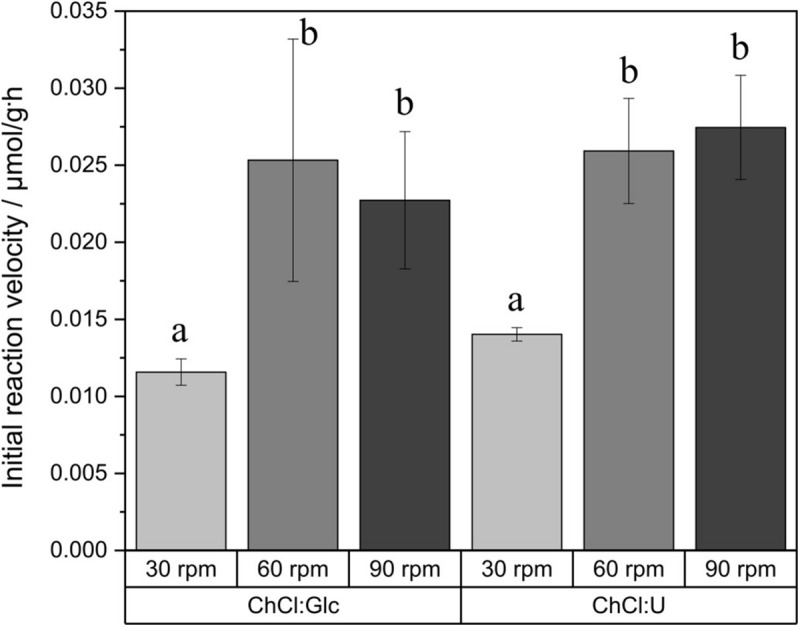
Initial reaction velocity in relation to the agitation rate. Glucose monodecanoate was determined directly by product quantification. Reaction conditions: 0.5 M vinyl decanoic acid, 50°C. a and b shows statistically significant differences. ChCl:Glc (ChCl:Glc:water, 5:2:5, n:n:n) and ChCl:U (ChCl:U, 1:2, n:n, 5% water).

To examine the effect of the high viscosity of DES on external mass transfer, an experiment with different agitation rates (30, 60, and 90 rpm) was set up and the initial reaction velocity was analyzed (Figure 3). There was neither a statistical difference concerning the initial reaction velocity between the two investigated DES nor a significant interaction between the type of DES and the agitation rate. For both DES, there was significant increase in the initial reaction velocity with increasing agitation rate. However, higher agitation rates than 60 rpm had no effect on the reaction rates.

### Influence of Fatty Acid Concentration

**FIGURE 4:**
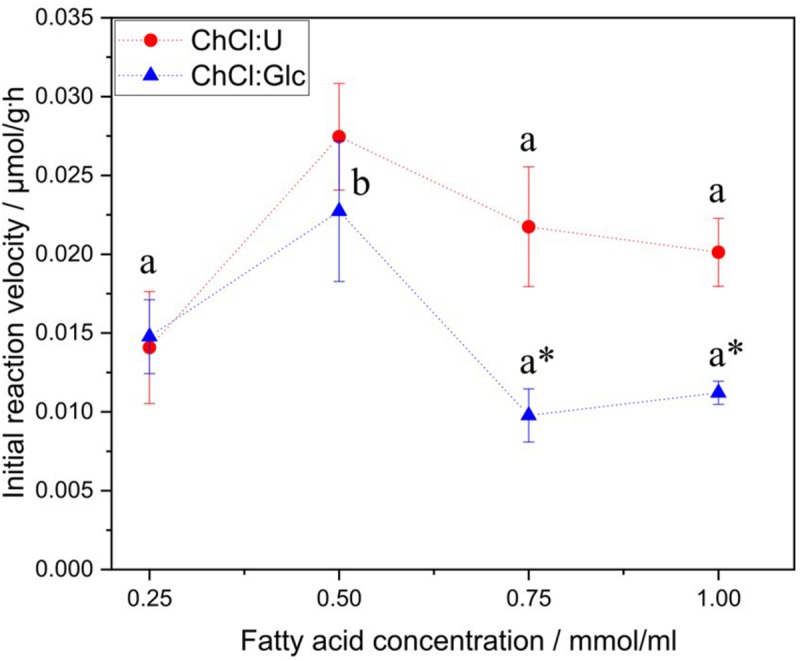
Impact of different fatty acid concentrations on the initial reaction velocity in ChCl:U (ChCl:U, 1:2, n:n, 5% water) and ChCl:Glc (ChCl:Glc:water, 5:2:5, n:n:n). Glucose monodecanoate was determined directly by product quantification. Reaction conditions: 90 rpm, 50 C. a and b shows statistically significant differences between the fatty acid concentrations. * shows statistically significant differences between the two DES.

Investigations of the initial reaction velocity in relation to the fatty acid concentration revealed a significant increase in the initial reaction velocity, with an increase in fatty acid concentration from 0.25 to 0.5 mol/L (Figure 4). However, a further increase in the fatty acid concentration did not cause any increase in the initial reaction velocity; rather, it resulted in a reduced initial reaction rate at fatty acid concentrations higher than 0.5 mol/l for both DES.

### Influence of Fatty Acid Distribution

**FIGURE 5:**
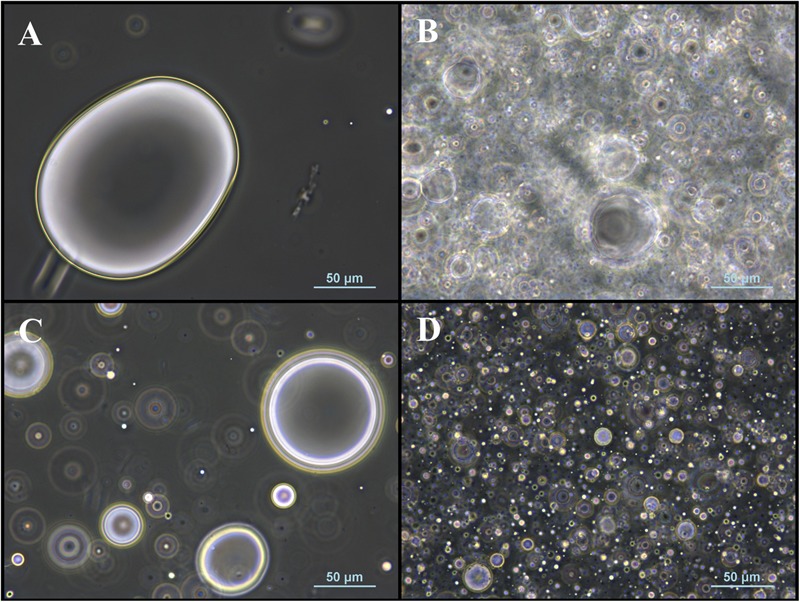
Microscopic pictures of untreated and sonicated fatty acid-DES emulsions. **(A)** Untreated fatty acid-ChCl:Glc emulsion, **(B)** sonicated fatty acid-ChCl:Glc emulsion, **(C)** untreated fatty acid-ChCl:U emulsion, and **(D)** sonicated fatty acid-ChCl:U emulsion. Fatty acid concentration in all fatty acid-DES emulsion was 0.5 mmol/mL. The images were obtained using phase contrast and a Nikon Eclipse E200 microscope.

**FIGURE 6:**
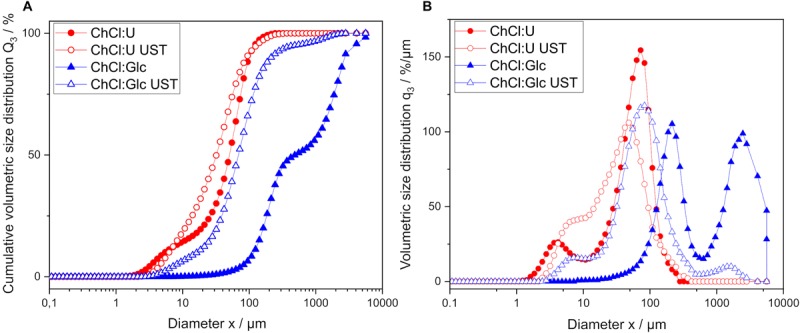
Impact of ultrasonic pretreatment (UST) on droplet size distribution of fatty acid-DES emulsions. **(A)** The cumulative volumetric size distribution Q_3_ and **(B)** the volumetric size distribution q_3_.

The effect of an ultrasonic treatment on fatty acid distribution in fatty acid-DES emulsions and on the resulting reaction rates was investigated. Optical microscopic analysis of fatty acid-DES emulsions showed smaller and more homogenously distributed fatty acid droplets after sonication for both DES. The droplet sizes obtained were smaller for ChCl:U than for ChCl:Glc (Figure 5). The untreated fatty acid-DES emulsions showed bimodality (Figure 6). The cumulative volume distribution of fatty acid-DES emulsions shifted toward smaller droplet diameters after sonication treatment, and bimodality was reduced. Significant differences in the mean droplet size x_50,3_ were determined between the two investigated DES as well as between untreated and sonicated DES. There was reduction in the mean droplet size x_50,3_ by sonication for both DES: for ChCl:U, from 54 ± 7 μm to 35 ± 2 μm, and for ChCl:Glc, from 464 ± 250 μm to 51 ± 13 μm.

**FIGURE 7:**
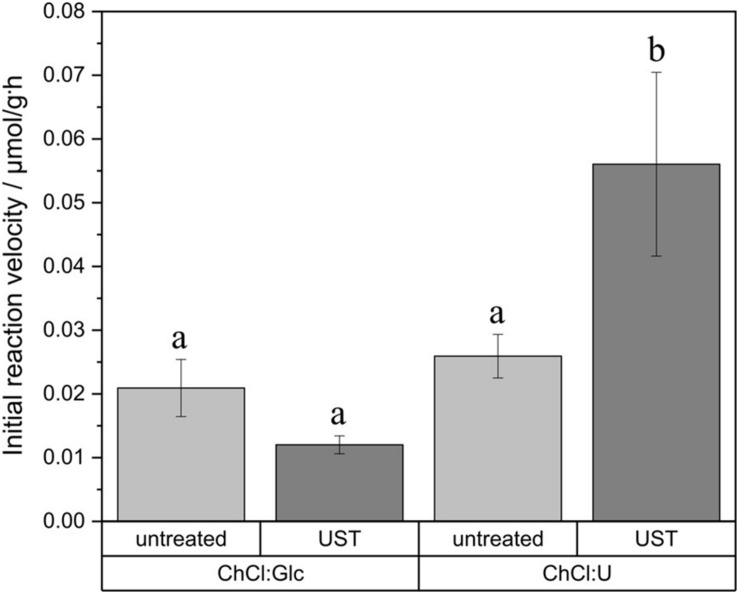
Impact of ultrasonic pretreatment (UST) on initial reaction velocity in ChCl:Glc (ChCl:Glc:water, 5:2:5, n:n:n) and ChCl:U (ChCl:U, 1:2, n:n, 5% water). Glucose monodecanoate was determined directly by product quantification. Reaction conditions: 0.5 M vinyl decanoic acid, 50°C, 90 rpm. a and b shows statistically significant differences.

Statistical analysis of initial reaction velocity in relation to ultrasonic pretreatment revealed a significant difference between ChCl:U and ChCl:Glc (Figure 7). The ultrasonic pretreatment had an influence on the initial reaction velocity but only for ChCl:U. The initial reaction rate of ChCl:U was significantly accelerated from 0.026 ± 0.003 μmol glucose monodecanoate/g DES ⋅ h to 0.056 ± 0.014 μmol/g ⋅ h. The glycolipid yield after 24 h synthesis in ChCl:U was increased by ultrasonic pretreatment from 0.15 ± 0.029 μmol/g to 0.57 ± 0.029 μmol/g.

## Discussion

The developed direct product quantification method enables the separation and quantification of monoesters and multiple esterified products, as well as educts which is more advantageous than the indirect analysis via substrate consumption. In the studies by Šabeder et al. (2006) and Bouzaouit and Bidjou-haiour (2016), an indirect quantification via the fatty acid amount, was performed. However, that quantification method can lead to an overestimation of the actual monoester formation, as substrate consumption offers no differentiation between monoesters and multiple esterified products. In contrast, direct quantification enables the identification and monitoring of different reaction phases, i.e., the transition from formation of monoesters to di- or polyesters. In the studies where a direct glycolipid quantification via HPLC was performed, refractive index detection was used (Castillo et al., 2003; Lin et al., 2015; Zhao et al., 2016). However, refractive index detection is incompatible with gradient elution, but ELSD detection allows for this (Clement et al., 1992; Swartz, 2010). In addition, ELSD detection has a higher sensitivity compared to refractive index detection, thus more suitable for low product concentrations (Clement et al., 1992; Hopia and Olialainen, 1993; Swartz, 2010).

Due to the viscosity of the investigated DES, it was not possible to directly inject the samples into the HPLC, so that either a dilution step must be performed to reduce the viscosity of the sample, as proposed by Zhao et al. (2016), or sample extraction must be performed. In the case of ChCl:Glc, an extraction is mandatory in order to avoid overloading the column with glucose and the associated poor separation of glucose and glucose decanoate. In addition, a strong dilution may conceal low concentration products. Since concentrations of 0.5 μmol/mL and lower were expected on the basis of preliminary studies and the findings of Zhao et al. (2016), a sample extraction approach was chosen in this study to overcome that issue.

To the best of our knowledge, there are no studies evaluating the extraction solvents for glycolipid extraction from DES. In the eighties, chloroform was still used as solvent for biosurfactant extraction from fermentation broth (Robert et al., 1989; McInerney et al., 1990). Later, EtAc started replacing chloroform as an extraction solvent (Koster et al., 1994; Siebenhaller et al., 2016). More recently, to extract polar and nonpolar lipids from microalgae, DMC is used as an alternative to traditional chloroform extraction (Lee et al., 2013; Tommasi et al., 2017). On this account, these three solvents have been chosen to be investigated. The more similar a certain solvent and the compound of interest, the better the solubility of that particular solvent is. To quantitatively evaluate this, and interpret the observed results, solubility parameters, e.g., Hansen solubility parameters, are a useful tool. Hansen solubility parameters describe solvent properties like nonpolar interaction, dipolar interaction and hydrogen bonding interaction (Hansen, 2004). Hansen parameters for chloroform indicate increased nonpolar interactions than ethyl acetate and DMC, as well as lower dipolar and hydrogen bonding interactions (Rahmalia et al., 2014). Thus, the theoretical and experimental results coincide, since glycolipids are polar molecules and chloroform presents the worst results of the three solvents investigated. DMC has higher Hansen parameters for dipolar and hydrogen bonding interactions than EtAc (Rahmalia et al., 2014). As DMC is less efficient in glucose monodecanoate extraction than EtAc, it is concluded that the amphiphilic glucose monodecanoate is less polar than DMC and therefore better extractable with less polar EtAc.

Furthermore, safety and toxicology are important aspects when selecting an extraction solvent. For the classification of solvents as green solvents, the following aspects must be considered: the entire life cycle of the solvents, safety in handling, health hazards and environmental compatibility (Jessop, 2011; Prat et al., 2015). EtAc and DMC are classified as recommended solvents while chloroform is classified as highly hazardous and should be avoided even in the laboratory (Prat et al., 2015). The investigation of commonly applied chloroform, EtAc, and uncommon DMC as solvents for glycolipid extraction from DES showed that the green solvents EtAc and DMC have a statistically higher efficiency than the harmful commonly applied chloroform. Based on extrapolation from the yield of sonicated ChCl:U experiments, 894 mL EtAc, 1307 mL DMC or 2429 mL CHCl_3_ would be necessary for the extraction of 1 g glucose monodecanoate. Therefore, it is advisable to use EtAc, which was confirmed as the most efficient extraction solvent, or DMC for glycolipid extraction.

The measured viscosity of ChCl:Glc is in good accordance with the value reported by Dai et al. (2013). Viscosity of DES are 100 to 2000 times higher than those of organic solvents (Smith et al., 2014; Xu et al., 2017), and are therefore regarded as the major problem of DES applications since they may pose mass transfer limitations (Dai et al., 2013; Zhao et al., 2016). Instead, the results of the external mass transfer limitation test show that, at least in our process, viscosity is only a limiting factor for glycolipid synthesis at agitation rates below 60 rpm. At agitation rates of 60 rpm and higher, external mass transfer limitation due to the high viscosity of DES can be excluded, since the statistical analysis revealed no difference between the two differently viscous DES and there is no statistical difference in reaction velocities. These findings also apply for sonicated samples (Supplementary Figure S2). Therefore, it can be assumed that the mixing is sufficient and there is no external mass transfer limitation for non-sonicated samples, as well as for sonicated samples. Similar results with regard to external mass transfer in DES were described by Ülger and Takaç (2017), where methyl gallate synthesis in DES was not enhanced at higher agitation rates after a certain threshold. Moreover, the relationship between agitation rate and initial reaction velocity was also used as a measure of external mass transfer in organic media and in enzymatic synthesis of antibiotics (Zhang et al., 2004; Gonçalves et al., 2008).

Investigations of the influence of fatty acid concentration on the initial reaction velocity revealed an inhibiting effect of fatty acid concentrations higher than 0.5 mol/L. Similar findings were reported for glucosyl myristate synthesis in organic solvents (Moreau et al., 2004; Cauglia and Canepa, 2008). Besides, fatty acid inhibition of CalB was also reported for other transesterification reactions, e.g., fatty acid esters, acetoin fatty acid esters or citronellol laurate (Yadav and Lathi, 2004; Lopresto et al., 2014; Xiao et al., 2015). Esterification and transesterification reactions follow a ping pong mechanism, and fatty acids are inhibitors by forming non-productive complexes with the enzyme (Zaidi et al., 2002; Serri et al., 2006; Lopresto et al., 2014). This effect of fatty acids on esterification reactions was also observed for other lipases than CalB, e.g., *Candida rugosa* lipase or *Rhizopus oryzae* lipase (Zaidi et al., 2002; Serri et al., 2006; Salah et al., 2007).

Once the existence of an external mass transfer limitation due to the high viscosities of DES could be excluded and an appropriate fatty acid concentration was chosen, it was investigated whether the fatty acid accessibility is a limiting factor. Sonication was reported as an effective method for emulsification (Abismaïl et al., 1999) and was therefore selected as treatment for improving fatty acid distribution. Pandolfe (1981) reported a decrease in efficiency of sonication with increasing viscosity of the continuous phase; though at high viscosities (>0.1 Pa⋅s) this is no longer true. However, we observed significantly smaller mean droplet sizes for both the less and the more viscous DES upon sonication. Nevertheless, the resulting droplet size upon sonication was smaller for ChCl:U, the less viscous DES, than for ChCl:Glc. Remarkably, for the more viscous DES ChCl:Glc a considerably greater droplet size reduction was achieved (by 89%) compared to the less viscous ChCl:U (by 36%).

Ultrasonic pretreatment led to a statistically significant higher initial reaction velocity in ChCl:U, as well as to an improved overall yield, and to a cumulative volume size distribution, which shifted toward smaller fatty acid droplet size. Hence, fatty acid distribution can be assumed as a limiting factor. Despite the stark droplet size reduction in ChCl:Glc, the initial reaction rate did not increase significantly upon sonication. In contrast, for ChCl:U the initial reaction rate increased significantly upon sonication although the mean droplet size decreased only slightly. Therefore, the different performances of the two tested fatty acid-DES mixtures might more likely be the result of the differences in polarity of the DES than due to their different viscosities: according to the solvatochromic parameter E_*T*_^*N*^, ChCl:U (0.835) is less polar than ChCl:Glc (0.845) (Oh et al., 2019). Oh et al. (2019) also showed that lipase activity in the transesterification of benzylalcohol and vinylacetate is higher in DES containing urea as hydrogen bond donor than in more polar DES containing glucose as hydrogen bond donor. An influence of solvent polarity on transesterification reactions was also reported for organic solvents and ionic liquids (IL). In organic solvents, the reaction yield in glycolipid synthesis with butanone as solvent is higher than with the more polar solvents acetone and t-butanol (Šabeder et al., 2006; Bouzaouit and Bidjou-haiour, 2016). Various IL investigations have shown that a compromise between highly hydrophilic ILs for good sugar solubility and highly hydrophobic IL for good fatty acid solubility is needed to achieve good conversion rates (Lin et al., 2015). Thus, both literature and the reported experimental results show that the polarity of the solvent is another factor that plays a role in glycolipid synthesis in DES. Besides the fatty acid, the alcohol substrate is also reported to act as an inhibitor on enzyme activity in esterification reactions by forming dead-end complexes (Zaidi et al., 2002; Lopresto et al., 2014). Therefore the glucose excess in ChCl:Glc might also contribute to the lower reaction yields compared to ChCl:U.

Also, other factors might be contributing to the different performances of the two DES, such as the different strength and nature of the hydrogen bonding network in the DES. The literature indicates that DES may contribute to lipase stability and activity by forming hydrogen bonds between DES and lipase, thereby stabilizing the tertiary structure of the enzyme (Kim et al., 2016; Elgharbawy et al., 2018; Oh et al., 2019). On the other hand, DES may also lower the lipase activity by destabilizing enzyme-substrate complexes or intermediate complexes (Kim et al., 2016; Elgharbawy et al., 2018; Oh et al., 2019). The stabilizing and destabilizing effects of hydrogen bonding between DES and lipase are in correlation with the nature of the DES, hydrogen bond acidity, and hydrogen bond basicity (Kim et al., 2016; Oh et al., 2019).

## Conclusion

In this study, limiting factors of glycolipid synthesis in DES were addressed and an optimization strategy was presented. For this, a quantification method consisting of an extraction method and an HPLC-ELSD measurement was developed, an external mass transfer limitation test was applied, the fatty acid concentration was optimized and the influence of droplet size distribution in fatty acid-DES emulsions on initial reaction velocities and glycolipid yield were investigated.

No differences in initial reaction velocities were observed for agitation rates of 60 rpm and higher. Therefore, it was shown that by using a proper agitation rate, an external mass transfer limitation in the investigated DES can be excluded. Results of the droplet size distribution measurements and the study of the initial reaction velocity of sonicated DES-fatty acid emulsions revealed that the fatty acid distribution is a limiting factor for glycolipid synthesis in ChCl:U. By applying a sonication treatment, the glucose decanoate yield of the enzymatic synthesis in ChCl:U was increased fourfold. However, despite obtaining a droplet size reduction, the initial reaction velocity of the more polar DES, ChCl:Glc, was not increased upon sonication. The effect of the polarity of DES on enzymatic glycolipid production should therefore be further addressed in a following study. In addition, alternative processes to sonication should be investigated as well as the use of adjuvants, both with the aim of reducing droplet size distributions. This will make the process more efficient and economical and will eventually make it possible to scale up.

## Data Availability Statement

The datasets generated for this study are available on request to the corresponding author.

## Author Contributions

RH planned and conducted the experiments. RH and BB performed the droplet size distribution measurements, supervised by US. RH analyzed the results and wrote the manuscript with conceptual advice of CS and KO. All authors critically read and approved the final manuscript.

## Conflict of Interest

The authors declare that the research was conducted in the absence of any commercial or financial relationships that could be construed as a potential conflict of interest.

The reviewer, TH, declared a past co-authorship with the authors to the handling Editor.
