# Supplementary figures and images for: Optimization of Glycolipid Synthesis in Hydrophilic Deep Eutectic Solvents
Source: Front Bioeng Biotechnol. 2020 May 5;8:382. doi: 10.3389/fbioe.2020.00382 (PMC7214929; doi:10.3389/fbioe.2020.00382)

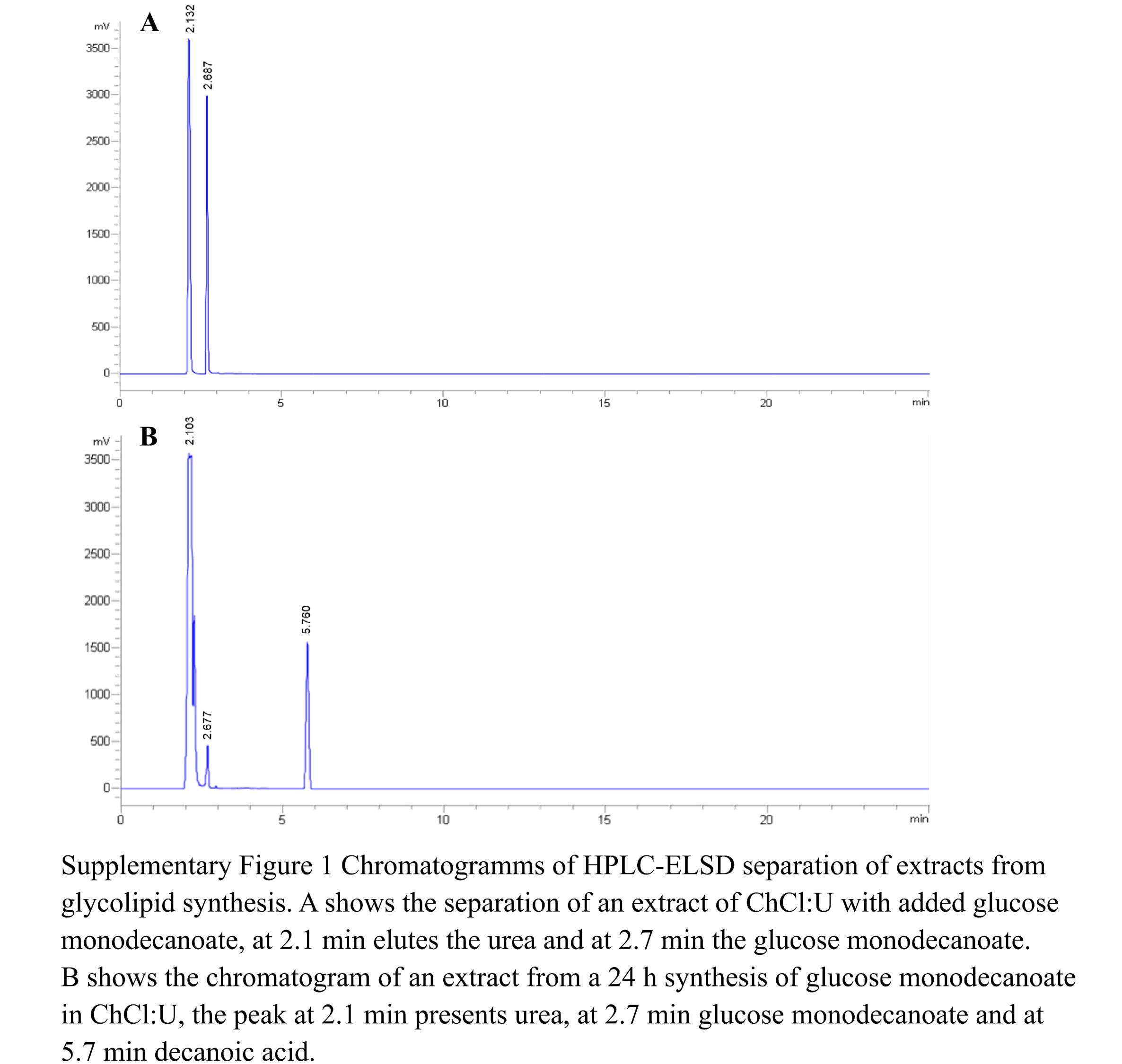

Supplement: Supplementary file 1 [file Image_1.JPEG]

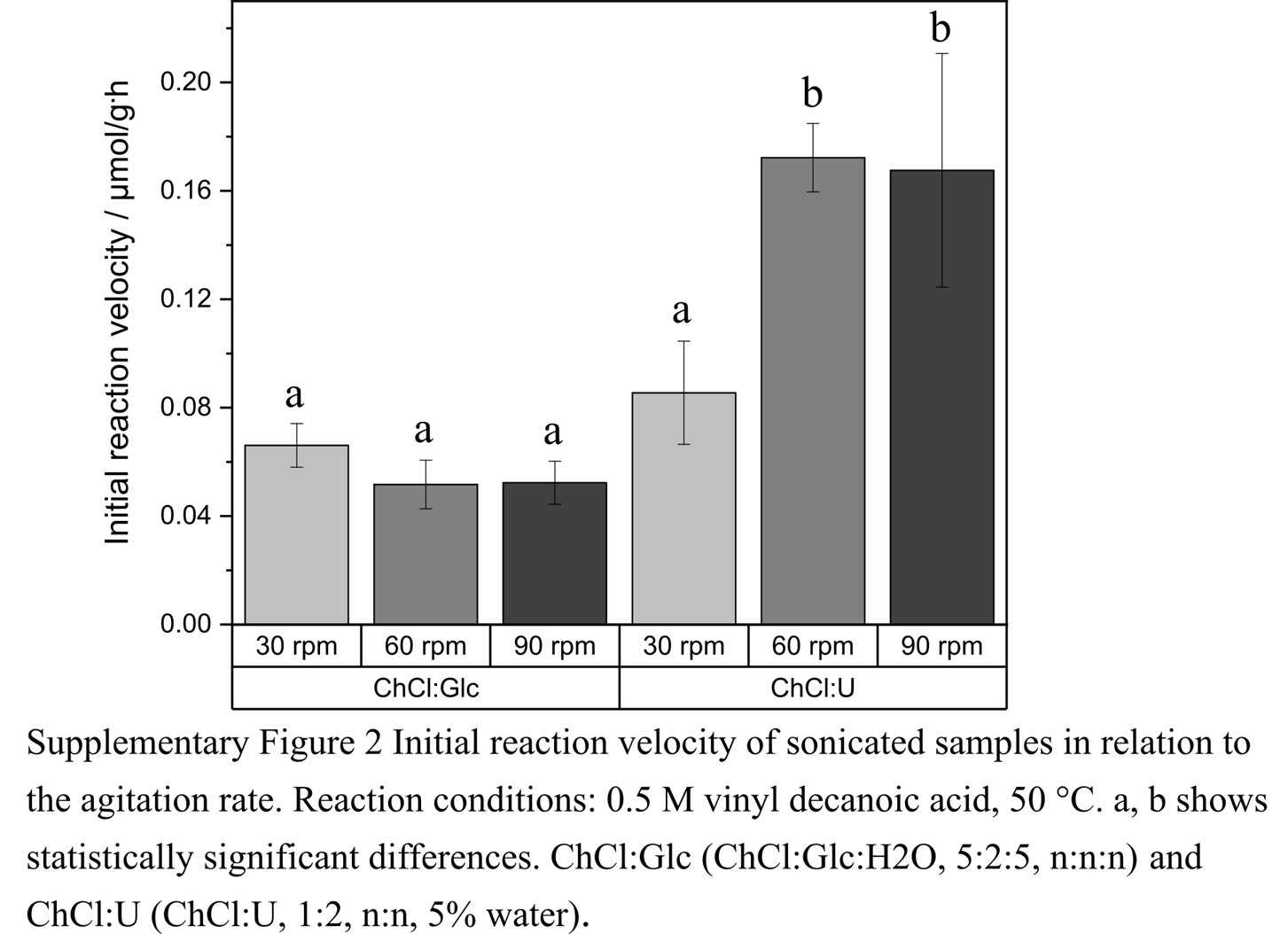

Supplement: Supplementary file 2 [file Image_2.JPEG]
